# Cultivating Anti-Racism Allies in Academic Medicine

**DOI:** 10.1089/heq.2022.0024

**Published:** 2023-04-12

**Authors:** Cassandra D.L. Fritz, Shirlene Obuobi, Monica E. Peek, Monica B. Vela

**Affiliations:** ^1^ Division of Gastroenterology, Diversity, Equity, and Inclusion for Internal Medicine Residency Program, Department of Medicine, Washington University School of Medicine, St. Louis, Missouri, USA.; ^2^ Division of Cardiology, Department of Medicine, The University of Chicago, Chicago, Illinois, USA.; ^3^ Section of General Internal Medicine, Chicago Center for Diabetes Translation Research, MacLean Center for Clinical Medical Ethics, The University of Chicago, Chicago, Illinois, USA.; ^4^ Department of Medicine, The Hispanic Center of Excellence, University of Illinois College of Medicine, Chicago, Illinois, USA.

**Keywords:** anti-racism, allies, academic medicine, microaggressions, macroaggressions, acts of omission

## Abstract

Racial microaggressions, racially based remarks, or actions that negatively impact marginalized physicians of color (Black, Latino/a/x, and American Indian/Alaskan Natives) often go unaddressed. This article provides four strategies for how individuals and institutions can engage in anti-racism allyship: (1) be an upstander during microaggressions, (2) be a sponsor and advocate for physicians of color, (3) acknowledge academic titles and accomplishments, and (4) challenge the idea of a “standard fit” for academic faculty and research. Skills in academic allyship should be taught to *all* physicians throughout the educational continuum to mitigate feelings of isolation that racialized minority physicians frequently experience.

United States events of state-sanctioned violence (i.e., police brutality) have rekindled national discussions about institutional and interpersonal racism. The attention has predominantly focused on the impact of racism on patients and the health and well-being of marginalized populations. There is growing literature and concern about institutional and interpersonal racism directed explicitly against physicians,^1^ especially Black and Latino/a/x physicians.^2,3^ Institutional and interpersonal racism impact the learning environment, organizational culture, and physician health and well-being.^4^

Black and Latino/a/x physicians with other socially marginalized identities based on gender or sexual identity are at additional risk for discrimination. This intersectionality has placed physicians who are female *and* Black or Latina at amplified risk of interpersonal racist and gender-biased interactions with patients, colleagues, and division/departmental leadership, likely contributing to lower promotion rates^5^ and increased attrition from academic medicine.^6^

## Inclusive Academic Environments Are Essential

To promote health equity, academic excellence, and scientific innovation, academic medical centers should focus on building diverse and inclusive environments.^7^ Given the significant costs interpersonal racism has on physicians' of color physical, emotional, and mental health,^8^ addressing issues of interpersonal racism and bias are essential. Racialized minorities compose a small percentage of the faculty physician workforce [Black (3.6%), Latino/a/x (3.2%), and American Indian/Alaskan Natives (0.1%),^9^ referred to as marginalized physicians of color throughout this article]. Yet, marginalized physicians of color are much more likely to experience racism and microaggressions^2,3,8,10^ while navigating predominately White academic institutions.^10^

As marginalized physicians cannot build inclusive academic climates on their own, cultivating academic allies is currently an underdeveloped strategy. Academic allies are colleagues committed to transforming their support into action. Learning and adequately using ally actions^11^ challenge colleagues to grow from passive bystanders to active participants. Allies are essential, partly because interpersonal racism can be subtle and may not rise to the level of reportable discrimination. Yet, these interactions, including professional gaslighting, are nonetheless harmful and contribute to the lack of inclusive diverse academic environments.

Academic centers will not achieve inclusive diverse work environments without addressing the effects of these harmful racially based interactions. Interpersonal racism, particularly racial microaggressions in the workplace, occurs frequently.^12,13^ *Racial microaggressions* are common verbal, behavioral, or environmental indignities. Whether intentional or unintentional, microaggressions communicate hostile, derogatory, or harmful racial slights and insults toward physicians of color.^12^ Sue et al. created a taxonomy of microaggressions that includes three broad categories: microinvalidation, microinsult, and microassault.^12^ Microinvalidations are comments that exclude or negate the experience or feelings of a marginalized person. A microinsult is an insensitive comment that demeans a marginalized person's heritage or identity. A microassault is an explicit, usually conscious, verbal, or nonverbal, attack on a marginalized person.

In contrast to a microassault, *macroaggressions*, the most explicit form of interpersonal racism, are overt verbal or physical racist assaults conducted in a public forum to purposefully create a hostile environment with lasting consequences to the marginalized person.^14^ Macroaggressions also provide the framework for individual microaggression to appear less consequential. However, the impact of a single microaggression can be felt acutely, and the impact of repeated microaggressions culminates over time. Both microaggressions and macroaggressions negatively impact the cognitive load, mental health, and productivity of Black physicians.^15^

## Micro-/Macroaggressions Have a Significant Cognitive Cost

The costs of microaggressions and macroaggressions are far-reaching and have been found to affect the cognitive function of all learners, but with different effects among White and Black trainees. A study assessing cognitive load in relation to micro-/macroaggression demonstrated White students were most affected by viewing macroaggressions, whereas Black students had the most significant cognitive load impairment after witnessing microaggressions.^16^

White colleagues may be less able to identify microaggressions and be less aware of the profound impact microaggressions have on marginalized physicians of color. Therefore, academic allies need to listen, learn, and believe the experience of marginalized physicians of color, so they know when and how to intervene as an ally. In addition, academic allies need to be mindful that certain marginalized groups (i.e., Black physicians) may require different levels of action from allies due to the historic type and frequency of microaggressions experienced by particular marginalized groups.

In addition to addressing *interpersonal* racism, allies are crucial because they can help address *institutional racism*, defined as differential access to goods, services, and power based on race.^17^ Within academic medicine, institutional racism includes differential access to information, advanced educational opportunities, resources, and having the power to influence decisions, leaders, and policies. This type of racism compounds societal norms, unearned privileges, and inaction in the face of societal oppression of marginalized groups.

## Building Inclusive Environments Requires Academic Allies in Anti-Racism

Because of the pernicious nature of interpersonal and institutional racism and its effects on all learners, particularly marginalized physicians of color, it will take a combined effort from physicians of color and their White physician allies to truly address racism within academic medicine. We provide four strategies for physicians to become anti-racism allies in academic medicine.

### Be an upstander in encounters of workplace racial discrimination

**FIG. 1.**
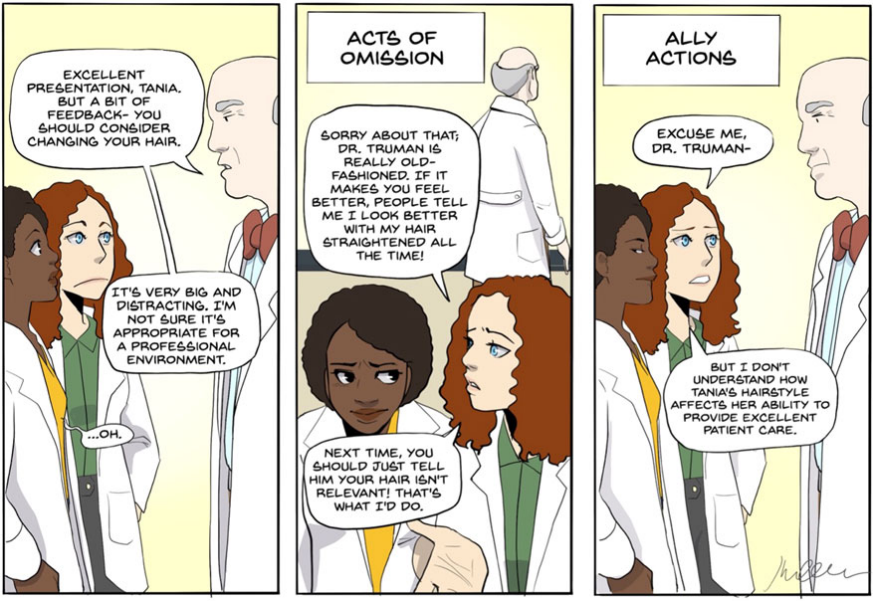
Difficult conversations: Act of Omission versus Ally Action. Comic description of the difference between an act of omission and an ally action. Drawn by Dr. Shirlene Obuobi @ShirlyWhirlMD.

Physicians of color need allies, specifically upstanders, who are committed to proactively engaging during acts of micro-/macroaggressions. They need colleagues invested in socially mindful interactions and conversations (Table 1). To do this, they need colleagues who do not continually worry about saying the “right thing” but colleagues who are willing to dismantle conversations rooted in racism (Fig. 1). Academic allies are built from colleagues who are eager to listen to the stories of physicians of color, willing to shoulder the burden of these experiences, and ready to work *with marginalized physicians of color* to address injustice. Upstanders create safe and inclusive space in academic encounters. In contrast, being a silent bystander is an act of omission—acts of omission further burden physicians of color and increase feelings of isolation in academic medicine. In the most extreme terms, acts of omission have been described as (possibly unintended) interpersonal forms of racism.^17^

**Table 1.** Difficult Conversations: Acts of Omission Versus Ally Actions

Example interaction	Acts of omission	Ally actions	Implication
Microinvalidation A Black physician is upset due to a patient refusing care from him/her due to his/her race.^24^ White colleagues then comment that they are sure the patient did not mean to be rude, offensive, or racist as they see the patient without the Black physician.	Negating the Black physician's feelings about the racist interaction is a microinvalidation and excluding them from the patient encounter leads to feelings of isolation. By not involving the Black physician in the encounter, the White colleague misses a critical opportunity to be an upstander.	An ally would inform the patient of the roles and necessary involvement of all providers on the team. An ally actively involves and supports the Black colleague during the encounter.	By using your platform of privilege in the patient care space: (1) You support the Black colleague's pursuit of medical education in all situations. (2) You model to other team members how to handle difficult and racist patients.
Microinsult You witness a White colleague “complement” a physician of color on how articulate they are.	The act of omission is not intervening during the interaction. Most acts of omission are also followed with words of “advice” to the physician of color after the microaggression. Stating, “I think you should report their inappropriate comment,” further burdening the physician of color.	An ally would be willing to ask uncomfortable questions to address the microinsult in the moment, stating, “why are you surprised by his/her speech, intellect, or skill?”	(1) You use your privilege to normalize the use of questions to confront bias and assert that the comments are inappropriate. (2) You model allyship by shouldering the burden of this moment with your racialized minority colleague.
Microassault A Black physician is “released” from a committee for addressing race/culture in an academic setting.	The White physician not leveraging their privilege or position of power to intervene on the committee decision is an act of omission. A White colleague “supporting” a physician of color behind closed doors stating, “the committee's actions were wrong and rooted in racism” does not actually provide any support or change the circumstance for the Black colleague.	An ally would refuse to serve on the committee that excluded the minority colleague. An ally would engage in difficult conversations regarding the process of appointment and dismissal from said role or position.	By asking difficult yet directed questions to the majority: (1) You highlight that the process, or lack thereof, may only benefit the majority. (2) You shed light on the power of the silent majority, and you will encourage others to acknowledge the racist and bias infrastructure.

### Actively using your privilege to look for equity opportunities for physicians of color

Allies need to understand the power of their privilege to address bias and racism. For those who endeavor to be academic allies, enter every room or meeting, and ask, “Who is missing in this room?,” “Am I making decisions that a diverse and inclusive team would better inform?,” and “Who is this policy benefitting or excluding?” Marginalized physicians of color need allies who recognize their rightful seats at the table and are willing to work to create those seats. One successful strategy has been for allies to make their participation on high-impact committees or invited panels contingent upon sufficient representation of diverse colleagues. Allies can suggest colleagues who are excellent candidates for these opportunities to serve alongside them or even take their place.

Allies using their platform of privilege to advocate for marginalized physicians of color will have a twofold effect. First, physicians of color will have increased access to spaces where they have traditionally been excluded. Second, White allies will benefit from favorable interactions with physicians of color, which has been shown to diminish implicit biases among majority trainees.^18^

### Acknowledge academic titles and accomplishments in public forums and official documents

Studies have shown that racialized minorities and women are less likely to be referred to as “doctor” (vs. their first name) in letters of recommendation and Grand Rounds introductions^19^ as compared with their White male counterparts. This implicit (or explicit) bias has negative implications for the career trajectories of marginalized individuals. It perpetuates social norms that devalue the academic accomplishments of physicians of color, particularly Black and Latina women. Building inclusive academic environments will require a commitment from allies to address their marginalized colleagues of color by their academic titles in public forums and official documents and work to create a culture in which their institutions routinely do the same.

### Challenge the idea of a “standard” fit for faculty and research: Embrace diverse people and ideas

The power of diversity is that individuals bring different lived experiences, ideas, and viewpoints to the table. Diverse work teams are more innovative, productive, and successful than homogenous teams with a monolithic way of thinking.^20^ Yet, the culture in academic medicine predominately values the status quo and stifles the presence and voice of racialized minorities, thus ultimately hindering patient-centered care^21^ and scientific innovation.^22^ Racial and gender-based diverse colleagues produce higher rates of novel scientific contributions; yet, they are less likely to have successful scientific careers compared with their White counterparts.^23^

Practices such as reviewing faculty candidates for “fit” into the existing institutional culture and relying on grant reviewers without content expertise to assess the merits of research proposals in areas such as health disparities, institutionalized racism, and community engagement should be rooted out. Our academic culture needs to change, and the structures that support institutional racism need to be dismantled and reimagined. The inaction and silence of colleagues, institutional leaders, and accrediting organizations regarding bias and structural racism have been deafening to marginalized physicians of color.

## Summary

If academic institutions want to cultivate inclusive diverse environments, addressing bias and counteracting the burden of interpersonal and institutional racism needs to be of the utmost priority. All physicians, across the pipeline, from medical students to faculty, would benefit from adopting intentional ally actions. Allyship training for physicians could mitigate occurrences of interpersonal racism in health care. Moreover, physicians need to understand that acts of omission further isolate and only increase the burden on marginalized physicians of color.

Allies and institutions who support allyship must have the courage to help change a system whose foundation is built on structural racism that perpetuates modern-day inequities for physicians of color. Individual anti-racism ally strategies include being an upstander for a marginalized physician of color during micro-/macroaggressions, actively using privilege to provide equitable access to academic opportunities, and appropriately acknowledging physicians of color in public forums. On an institutional level, diverse people and ideas should be welcomed, supported, and celebrated as an approach for academic institutions to remain innovative and become more inclusive. These strategies could make substantial improvements in the lives and well-being of *all* physicians, particularly marginalized physicians of color.
